# Low serum apolipoprotein A1 level predicts poor prognosis of patients with diffuse large B-cell lymphoma in the real world: a retrospective study

**DOI:** 10.1186/s12885-024-11818-5

**Published:** 2024-01-12

**Authors:** Xiaoling Huang, Ying Wang, Zhenyu Huang, Xuzheng Chen, Qiuyan Lin, Haobo Huang, Liping Fan

**Affiliations:** 1 Department of Blood Transfusion, Fujian Medical University Union Hospital, Gulou District, Fuzhou City, 350001 Fujian Province China; 2 School of Food and Bioengineering, Fujian Polytechnic Normal University, Fuqing County, Fuzhou City, 350300 Fujian Province China; 3 Academy of Integrative Medicine, Fujian University of Traditional Chinese Medicine, Fuzhou City, 350122 Fujian Province China; 4 Fujian Key Laboratory of Integrative Medicine on Geriatrics, Fuzhou City, 350122 Fujian Province China

**Keywords:** Apolipoprotein A1, DLBCL, Prognostic factor

## Abstract

**Background:**

Apolipoprotein A1 (ApoA1) is a member of the apolipoprotein family with diverse functions. It is associated with the pathogenesis and prognosis of several types of tumors. However, the role of serum apolipoprotein A1 (ApoA1) in the prognosis of patients with diffuse large B-cell lymphoma (DLBCL) remains unclear. This study aimed to elucidate its influence on clinical outcomes in patients with DLBCL.

**Methods:**

We retrospectively analyzed a cohort of 1583 consecutive DLBCL patients admitted to the Fujian Medical University Union Hospital between January 2011 and December 2021. 949 newly diagnosed DLBCL patients who met the inclusion criteria were enrolled for statistical analysis. Receiver operating characteristic curve analysis was performed to determine the optimal cut-off value for serum ApoA1 levels for prognostic prediction among patients with DLBCL. The correlations between serum ApoA1 levels and clinical and laboratory parameters were analyzed. Prognostic significance was analyzed using univariate and multivariate Cox proportional hazards models.

**Results:**

Newly diagnosed patients with DLBCL demonstrated low serum ApoA1 levels (< 0.925 g/L), had more B symptoms, higher levels of serum lactate dehydrogenase (LDH) (>upper limit of normal), poorer performance status (Eastern Cooperative Oncology Group score of 2–4), higher percentage of advanced stage and non-germinal center B-cell (non-GCB) subtype, more cases of > 1 extranodal site, higher International Prognostic Index (IPI) score (3–5), and higher incidence of relapse or refractory diseases compared with those with high serum ApoA1 levels (≥ 0.925 g/L). Low serum ApoA1 levels were an independent adverse prognostic factor for overall survival (OS) but not progression-free survival (PFS).

**Conclusions:**

Low serum ApoA1 levels were associated with poor treatment response and inferior survival in newly diagnosed patients with DLBCL.

## Background

Diffuse large B-cell Lymphoma (DLBCL) is the most common type of aggressive B-cell non-Hodgkin’s lymphoma worldwide. Regarding the cell of origin, DLBCL can be classified as germinal center B-cell-like (GCB) and non-GCB cells by immunophenotypic detection. It can also be divided into activated B-cell-like, GCB, unclassified, or type III based on gene expression profiling. Regardless of cell origin, the standard first-line treatment for DLBCL is R-CHOP, which comprises the anti-CD20 monoclonal antibody rituximab plus cyclophosphamide, doxorubicin, vincristine, and prednisone. However, 40–50% of patients with DLBCL suffer from refractory disease or relapse throughout the administration of standard first-line treatment or follow-up [1–4]. Therefore, exploring the factors related to DLBCL pathogenesis may be helpful in the treatment and prognosis of patients.

Metabolic reprogramming is an important hallmark of tumor cells. Abnormal lipid metabolism has been reported in several types of tumors, which affects development and progression by regulating various oncogenic signal pathways [5–7]. Few studies have demonstrated that abnormal lipid characterization is associated with R-CHOP resistance in a mouse model of DLBCL and that serum free fatty acids, high-density lipoproteins (HDL), low-density lipoproteins, and triglycerides are associated with the prognosis of patients with DLBCL [8–12].

Apolipoprotein A1 (ApoA1), the main component of HDL, has been extensively studied in the context of atherosclerosis, thrombotic diseases, diabetes, and nervous system diseases. The findings of these studies showed that ApoA1 could bind to ATP-binding cassette transporter (ABCA1) of macrophages and endothelial cells, trigger cholesterol efflux from these cells, then reduce the production of inflammatory factors in these cells and the degeneration of macrophages, leading to protection of arterial wall. It could improve the integrity of endothelial barrier, inhibit the activity of von Willebrand factor (vWF) activity and increase the degradation of procoagulant factors, then reduce thrombosis. It could also bind to ABCA1 of β-cell, inhibit its’ apoptosis and increase insulin secretion, then improve glycaemic control. Furthermore, it could cross the blood-brain barrier, reduce amyloid beta (Aβ) deposition and levels, as well as neuroinflammation, exhibiting neuroprotective functions [13–15]. In recent years, an increasing number of studies on ApoA1 in cancers have been conducted [16–26]. However, the possible role of APOA1 in the pathogenesis and prognosis of DLBCL has been poorly studied [11, 12]. Therefore, we retrospectively analyzed the data of newly diagnosed patients with DLBCL at our center to explore the prognostic value of pretreatment serum ApoA1 levels.

## Methods

### Study population

We studied all consecutive patients with newly diagnosed DLBCL who were admitted to the Fujian Medical University Union Hospital between January 1, 2011, and December 31, 2021. All patients were diagnosed according to the World Health Organization (WHO) 4th revision classification of tumours of haematopoietic and lymphoid tissues. Newly diagnosed patients with DLBCL who were ≥ 14 years and received ≥ 4 cycles of chemotherapy or immunochemotherapy were included in this study. Patients diagnosed with primary mediastinal lymphoma, primary central nervous system lymphoma, human immunodeficiency virus infection, diabetes mellitus, or other malignancies were excluded from the study. Demographic information, clinical characteristics, and laboratory parameters, including age, sex, B symptoms, serum lactate dehydrogenase (LDH) levels, Eastern Cooperative Oncology Group (ECOG) score, Ann Arbor stage, extranodal disease sites, International Prognostic Index (IPI) score, Hans classification, serum ApoA1 levels, and therapeutic regimens, were obtained from medical records.

The same number of healthy individuals without metabolic and neoplastic diseases who presented in the aforementioned period were sex- and age-matched to the DLBCL group and assigned to the control group.

Approval for this study was obtained from the Ethics Committee of Fujian Medical University Union Hospital. As this study was a retrospective data analysis and did not affect patients’ treatments, written informed consent was not sought. This study was conducted in accordance with the Declaration of Helsinki.

### Sample collection and detection

Peripheral blood samples were collected from all patients within a week of primary therapy. Peripheral blood samples were collected from 949 healthy individuals who were included in the control group. All samples were collected from the patients and healthy individuals after overnight fasting.

Serum ApoA1 levels were measured by an immunoturbidimetry assay using a Roche cobas8000 automatic biochemical analyzer. Serum ApoA1 detection kit was purchased from Roche (Basel, Switzerland).

### Treatment and follow-up

All newly diagnosed patients with DLBCL included in this study had received ≥ 4 cycles of chemotherapy with or without a history of tissue biopsy or surgical excision. The therapeutic regimen comprised cyclophosphamide, doxorubicin, epirubicin, vincristine, and prednisone with or without rituximab. Patients who received fewer than three cycles of rituximab were assigned to the CHOP-like group; otherwise, patients were assigned to the R-CHOP-like group. The treatment response was evaluated according to the Revised Response Criteria for Malignant Lymphoma [27]. For disease progression or relapse, patients were treated with later-line regimens, as recommended by the NCCN guidelines [28–31].

Overall survival (OS) was calculated from the date on which the patient started treatment to the date of death from any cause or last follow-up. Progression-free survival (PFS) was calculated from the date on which the patient started treatment to the date of disease progression after first-line chemotherapy or immunochemotherapy, relapse, death, or last follow-up. The last follow-up date was February 10, 2023. Follow-up data were obtained from the clinical records or via telephone interviews.

### Statistical analyses

All statistical analyses were performed using SPSS software version 19.0 (IBM Corp., Armonk, NY, USA) and GraphPad Prism version 8.0 (GraphPad Corp., Boston, USA). The receiver operating characteristic (ROC) curve was used to determine the optimal cutoff value for serum ApoA1 levels. Categorical and continuous variables were compared using the χ^2^-test and t-test, respectively. Time-to-event data were analyzed using the Kaplan-Meier method. The log-rank test was used to compare the survival times of different groups. The Cox proportional hazards model was used for univariate analysis of the potential predictors of survival. The Cox regression model was used for the multivariate analysis of variables identified as significant prognostic factors in the univariate analysis. Statistical significance was defined as a two-sided *P*-value < 0.05.

## Results

### Baseline characteristics of patients with DLBCL

**Fig. 1:**
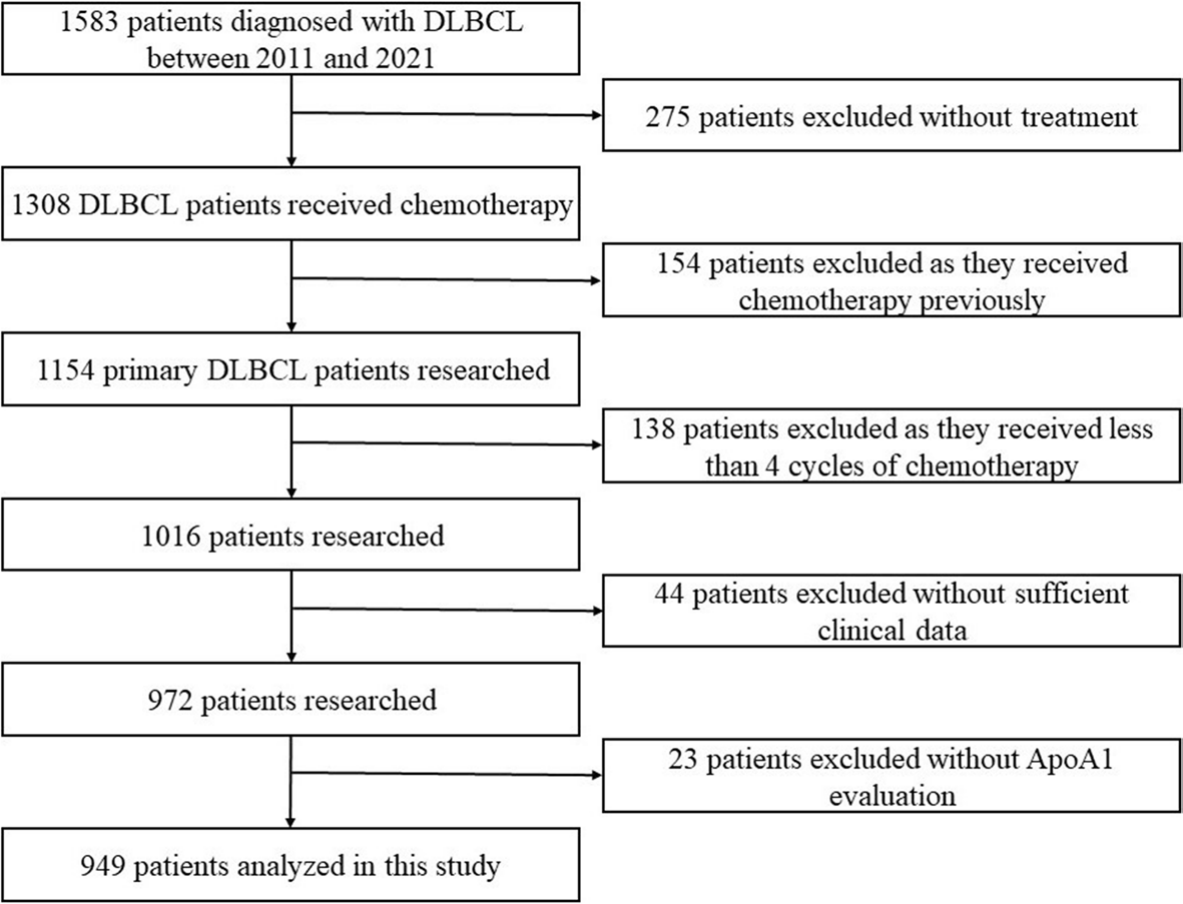
Flowchart showing the retrospective screening process for patients with DLBCL

There was a cohort of 1583 consecutive DLBCL patients admitted to the Fujian Medical University Union Hospital between January 2011 and December 2021. A total of 949 newly diagnosed patients with DLBCL who met the inclusion criteria were enrolled in this study based on the screening flowchart presented in Fig. 1.

In this cohort, the median age at diagnosis was 56 (range, 14–87) years. A total of 574 (60.48%) patients were male. Two hundred and twenty-six (23.81%) patients had B symptoms, 472 (49.74%) had higher (>upper limit of normal [ULN]) LDH levels, 200 (21.07%) had high Eastern Cooperative Oncology Group (ECOG) scores (2–4), and 626 (65.96%) had advanced Ann Arbor stages III (III–IV). High IPI scores (3–5) at diagnosis were found in 40.04% (380/949) of patients. The Hans classification of 50 patients could not be determined because their diagnostic tissue samples were insufficient. A total of 309 (32.56%) patients had the GCB subtype, whereas 590 (62.17%) had the non-GCB subtype. A total of 130 patients who received less than three cycles of rituximab were assigned to the CHOP-like regimen. The remaining 819 patients were assigned to the R-CHOP-like regimen.

### Serum ApoA1 levels in newly diagnosed patients with DLBCL and healthy individuals

**Fig. 2:**
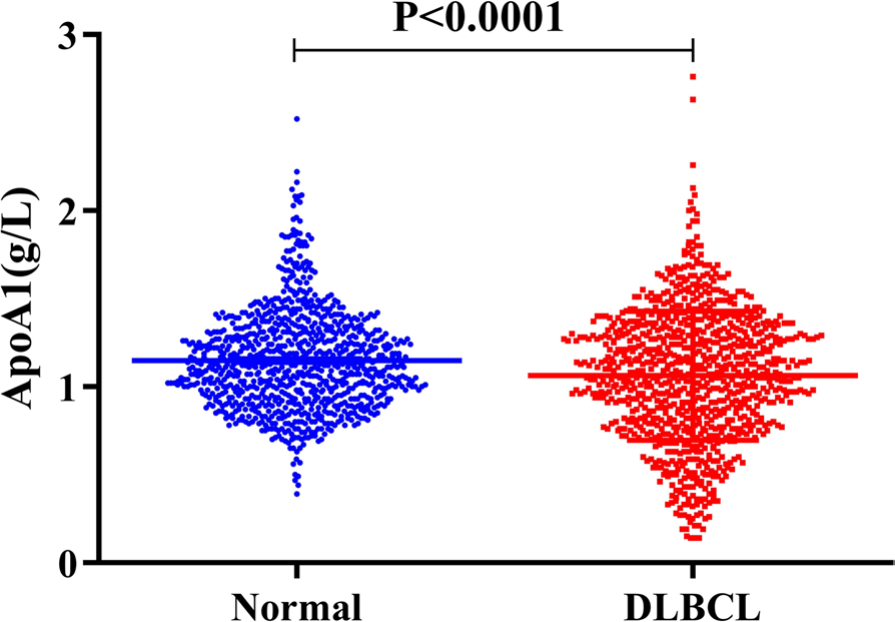
Serum ApoA1 levels in newly diagnosed patients with DLBCL and healthy individuals

The median value of serum ApoA1 levels in newly diagnosed patients with DLBC was 1.07 (range, 0.14–2.76) g/L. The median value of serum ApoA1 levels in healthy individuals was 1.12 (range, 0.39–2.52) g/L. Results showed that serum ApoA1 levels in newly diagnosed patients with DLBCL were significantly lower than those in healthy individuals (1.06 ± 0.011 g/L vs. 1.15 ± 0.010 g/L, *P* < 0.0001; Fig. 2).

#### Identification of the optimal cut-off value of serum ApoA1

**Fig. 3:**
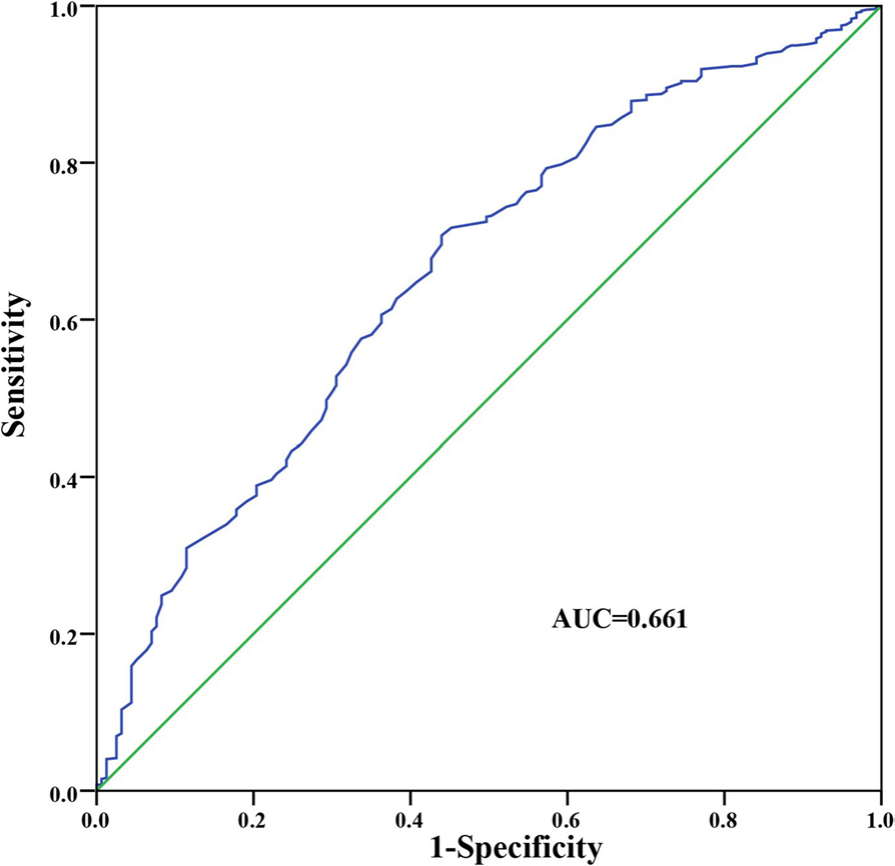
ROC analysis of pretreatment serum ApoA1 levels (AUC = 0.661; 95% CI: 0.614–0.707, *P* < 0.001)

According to the survival outcome of patients included, the optimal cut-off value of serum ApoA1 determined by the ROC curve was 0.925 g/L, with an area under the curve of 0.661 (95% confidence interval [CI]: 0.614–0.707, *P* < 0.001) (Fig. 3). According to the cutoff value, 629 (66.28%) and 320 (33.72%) patients were assigned to the high ApoA1 (≥ 0.925 g/L) and low ApoA1 groups (< 0.925 g/L), respectively.

### Association of clinical characteristics and treatment response with pretreatment serum ApoA1 levels

The clinical characteristics and treatment response as well as a comparison between the high ApoA1 and low ApoA1 groups are shown in Table 1. Compared with the high ApoA1 group, the low ApoA1 group had a significantly higher percentage of male patients (70.31% vs. 55.48%, *P* < 0.001), more B symptoms (44.69% vs. 13.20%, *P* < 0.001), higher levels of LDH (>ULN) (72.19% vs. 38.31%, *P* < 0.001), poorer performance status (ECOG score 2–4) (33.75% vs. 14.63%, *P* < 0.001), higher percentage of advanced stage (80.31% vs. 58.66%, *P* < 0.001), more cases in which there were more than one extranodal site (47.19% vs. 27.66%, *P* < 0.001), higher IPI (3–5) (58.13% vs. 30.85%, *P* < 0.001), and higher percentage of non-GCB subtype (70.79% vs. 63.16%, *P* = 0.025). Additionally, the low ApoA1 group had a higher incidence of relapse or refractory disease (37.50% vs. 25.12%, *P* < 0.001) than had the high ApoA1 group. There were no significant differences in age between both groups (*P* = 0.475).

**Table 1:** Association of clinical characteristics and treatment response with pretreatment serum ApoA1 levels

Parameters	Classification	ApoA1		P
		Low (*n* = 320)	Normal (*n* = 629)	
Age (years)^a^	> 60	114 (35.63%)	239 (38.00%)	0.475
	≤ 60	206 (64.38%)	390 (62.00%)	
Sex	Male	225 (70.31%)	349 (55.48%)	< 0.001
	Female	95 (29.69%)	280 (44.52%)	
B symptoms	Absent	177 (55.31%)	546 (86.80%)	< 0.001
	Present	143 (44.69%)	83 (13.20%)	
LDH	>ULN	231 (72.19%)	241 (38.31%)	< 0.001
	Normal	89 (27.81%)	388 (61.69%)	
ECOG score	0–1	212 (66.25%)	537 (85.37%)	< 0.001
	2–4	108 (33.75%)	92 (14.63%)	
Ann Arbor Stage	I–II	63 (19.69%)	260 (41.34%)	< 0.001
	III–IV	257 (80.31%)	369 (58.66%)	
Extranodal	>1	151 (47.19%)	174 (27.66%)	< 0.001
disease	≤ 1	169 (52.81%)	455 (72.34%)	
IPI	Low risk (0–1)	65 (20.31%)	291 (46.26%)	< 0.001
	Low-intermediate risk (2)	69 (21.56%)	144 (22.89%)	
	High-intermediate risk (3)	100 (31.25%)	121 (19.24%)	
	High risk (4–5)	86 (26.88%)	73 (11.61%)	
Cell of origin	GCB	85 (26.56%)	224 (35.61%)	< 0.001
	non-GCB	206 (64.38%)	384 (61.05%)	
	Undetermined	29 (9.06%)	21 (3.34%)	
Relapse/Refractory	Yes	120 (37.50%)	158 (25.12%)	< 0.001
	No	200 (62.50%)	471 (74.88%)	

*ApoA1 *Apolipoprotein A1, *LDH *Lactate dehydrogenase, *ULN *Upper limit of normal, *ECOG *Eastern Cooperative Oncology Group, *IPI *International Prognostic Index, *GCB *Germinal center B-cell-like. ^a^Mantissa of the two digits is the result. Mantissae of the three digits were added to 100%

### Univariate and multivariate analyses of potential prognostic factors for survival

The median follow-up of this cohort was 30.87 (range, 2.3–145.23) months. A total of 278 patients had relapsed or refractory disease, and 157 patients died.

**Fig. 4:**
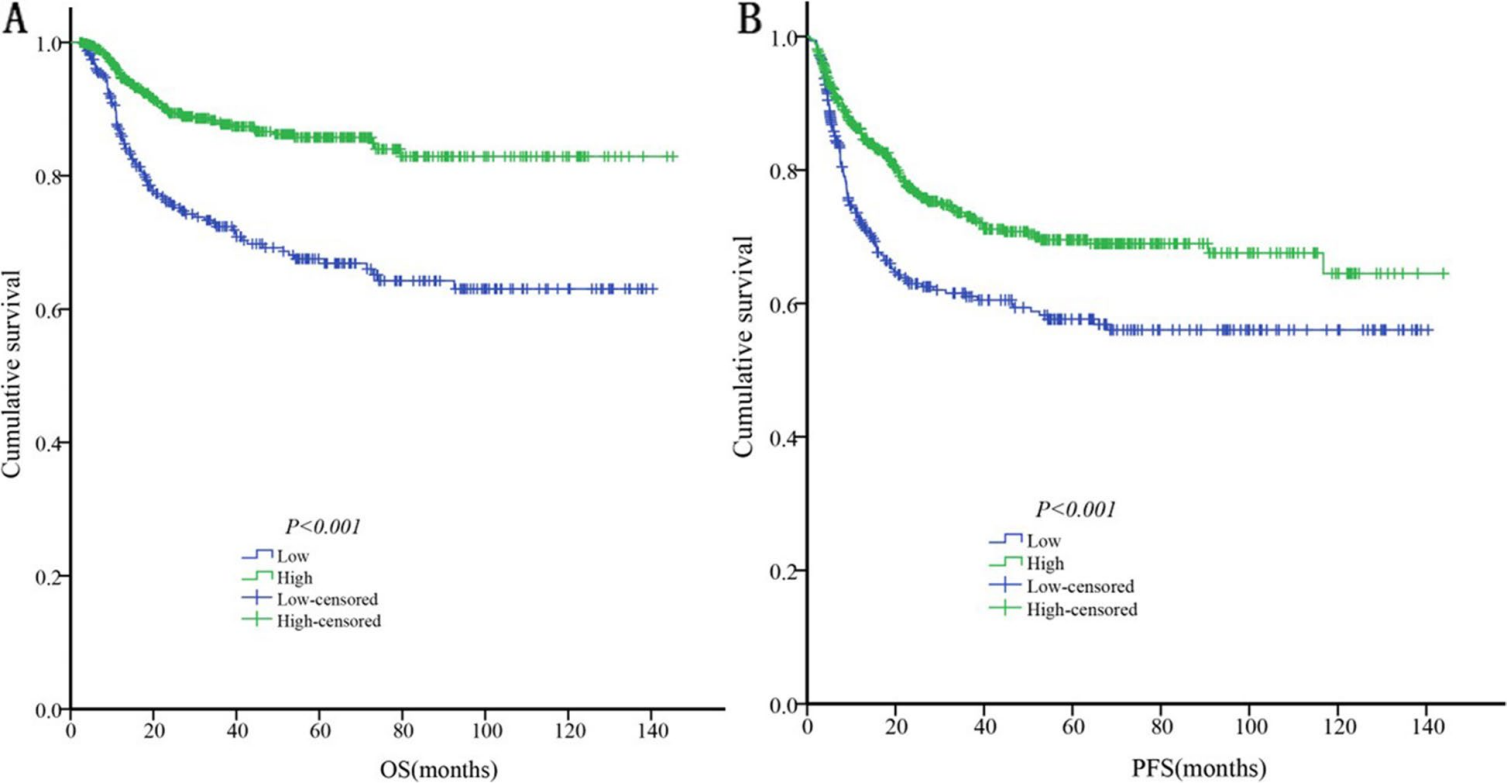
OS and PFS of newly diagnosed DLBCL patients according to pretreatment serum ApoA1 levels. **A:** OS; **B:** PFS

The Kaplan-Meier analysis demonstrated that the median OS and PFS in the low ApoA1 group were significantly lower (98.09 months vs. 125.48 months, *P* < 0.001; and 85.28 months vs. 103.13 months, *P* < 0.001, respectively) than those in the high ApoA1 group (Fig. 4).

In the univariate analysis, an age > 60 years (*P* = 0.014), advanced Ann Arbor Stage (III-IV) (*P* < 0.001), more than one extranodal site (*P* < 0.001), ECOG score ≥ 2 (*P* < 0.001), LDH > ULN (*P* < 0.001), presence of B symptoms (*P* < 0.001), low serum ApoA1 levels (*P* < 0.001), non-GCB subtype (*P* = 0.003), and non-utilization of rituximab (*P* < 0.001) were significantly associated with shorter OS. Multivariate analysis showed that an ECOG score ≥ 2 (*P* = 0.002), LDH > ULN (*P* = 0.001), low serum ApoA1 levels (*P* = 0.022), non-GCB subtype (*P* = 0.024), and non-utilization of rituximab (*P* < 0.001) were independent prognostic factors for shorter OS (Table 2; Fig. 4).

**Table 2:** Univariate and multivariate analysis of prognostic factors for OS in newly diagnosed patients with DLBCL

Parameters	Univariate analysis			Multivariate analysis		
	HR	95% CI	P	HR	95% CI	P
Sex (male vs. female)	1.26	0.91–1.75	0.165			
Age (>60 vs. ≤60)	1.49	1.09–2.04	0.014	1.25	0.88–1.76	0.215
Ann Arbor Stage (III–IV vs. I–II)	2.65	1.78–3.95	<0.001	1.39	0.87–2.23	0.166
Extranodal disease (>1 vs ≤1)	1.87	1.36–2.56	<0.001	1.09	0.76–1.55	0.645
ECOG score (2–4 vs. 0–1)	3.01	2.19–4.15	<0.001	1.83	1.24–2.70	0.002
LDH (>ULN vs. normal)	3.13	2.21–4.42	<0.001	1.93	1.29–2.88	0.001
B symptoms (present vs. absent)	2.27	1.65–3.13	<0.001	1.28	0.89–1.85	0.180
Serum ApoA1 (high vs. low)	0.39	0.28–0.53	<0.001	0.66	0.46–0.94	0.022
Subtype (non-GCB vs. GCB)	1.76	1.21–2.57	0.003	1.38	1.04–1.83	0.024
Regimen (CHOP-like vs. R-CHOP-like)	2.00	1.38–2.90	<0.001	2.21	1.51–3.23	<0.001

*CI* Confidence interval, *HR* Hazard ratio, *ECOG* Eastern Cooperative Oncology Group, *LDH* Lactate dehydrogenase, *ULN* Upper limit of normal, *ApoA1* Apolipoprotein A1, *GCB* Germinal center B-cell-like

In contrast, univariate analysis showed that an advanced Ann Arbor Stage (III-IV) (*P* < 0.001), more than one extranodal site (*P* < 0.001), ECOG score ≥ 2 (*P* < 0.001), LDH > ULN (*P* < 0.001), presence of B symptoms (*P* < 0.001), low serum ApoA1 levels (*P* < 0.001), non-GCB subtype (*P* < 0.001), and non-utilization of rituximab (*P* = 0.004) were significantly associated with shorter PFS. Multivariate analysis showed that advanced Ann Arbor Stage (III–IV) (*P* = 0.003), more than one extranodal site (*P* = 0.003), ECOG score > 2 (*P* = 0.011), LDH > ULN (*P* = 0.019), non-GCB subtype (*P* = 0.001), and absence of rituximab (*P* < 0.001) were independent prognostic factors for shorter PFS (Table 3; Fig. 4).

**Table 3:** Univariate and multivariate analysis of prognostic factors for PFS in newly diagnosed patients with DLBCL

Parameters	Univariate analysis			Multivariate analysis			
	HR	95% CI	P	HR	95% CI	P	
Sex (male vs. female)	1.11	0.87–1.42	0.408				
Age (> 60 vs. ≤60)	1.16	0.90–1.48	0.248				
Ann Arbor Stage (III–IV vs. I–II)	2.66	1.96–3.60	< 0.001	1.71	1.21–2.43	0.003	
Extranodal disease (>1 vs. ≤1)	2.18	1.71–2.77	< 0.001	1.49	1.14–1.95	0.003	
ECOG score (2–4 vs. 0–1)	2.00	1.54–2.60	< 0.001	1.44	1.09–1.91	0.011	
LDH (>ULN vs. normal)	2.15	1.68–2.75	< 0.001	1.41	1.06–1.87	0.019	
B symptoms (present vs. absent)	1.64	1.27–2.12	< 0.001	1.13	0.85–1.50	0.421	
Serum ApoA1 (high vs. low)	0.62	0.48–0.78	< 0.001	0.98	0.75–1.29	0.902	
Subtype (non-GCB vs. GCB)	1.76	1.33–2.33	< 0.001	1.47	1.18–1.84	0.001	
Regimen (CHOP-like vs. R-CHOP-like)	1.58	1.16–2.15	0.004	1.75	1.28–2.39	< 0.001	

*CI *Confidence interval, *HR *Hazard ratio, *ECOG *Eastern Cooperative Oncology Group, *LDH *Lactate dehydrogenase, *ULN *Upper limit of normal, *ApoA1 *Apolipoprotein A1, *GCB *Germinal center B-cell-like

## Discussion

Conventionally, the heterogeneity of immunology, cytogenetics, and molecular biology exists in DLBCL tissue, leading to heterogeneity in survival among patients with DLBCL. Currently, clinical studies have demonstrated that 40–50% of patients suffer from refractory or relapsed DLBCL after R-CHOP treatment [1–4]. Some studies have shown that intensified immunochemotherapy or addition of novel drugs to the R-CHOP regimen can improve the treatment response and prognosis [32–35]. Therefore, risk stratification of newly diagnosed patients with DLBCL is critical for the choice of treatment and, thus, affects outcomes. Thus far, indices for risk stratification of DLBCL include the IPI score and its derivatives, revised IPI and National Comprehensive Cancer Network IPI (R-IPI and NCCN-IPI). These indices comprise clinical factors including age, Ann Arbor stage, extranodal disease, ECOG score, and serum LDH level. However, none of these indices can efficiently distinguish high-risk patients from others [36]. Hence, it is necessary to identify other factors related to the development and progression of DLBCL, determine their role in the prognosis of patients with this disease, and establish useful prognostic indices.

Lipid metabolism-related genes and products have been reported to be involved in tumorigenesis and serve as biomarkers for tumor diagnosis and prognosis [5, 14, 37–39]. ApoA1 is a member of the apolipoprotein family that participates in reverse cholesterol transport and lipid metabolism. Basic studies have revealed that APOA1 plays a key role in the development and progression of solid tumors. In colorectal cancer, the expression of ApoA1 in tumor cells is associated with different metastatic tropisms [26]. In patients with squamous cervical cancer, APOA1 overexpression can reduce the sensitivity of cervical squamous cells to carboplatin by regulating the expression of STAT1, CD81, C3, and TOP2A [19]. In osteosarcoma, circ_0088212 exhibits a cancer-promoting role by regulating the miR-520 h/APOA1 axis [40]. In pancreatic ductal adenocarcinoma, ubiquitination and degradation of ApoA1 mediated by TRIM15 can promote the invasion and metastasis of pancreatic cancer cells. The APOA1-LDLR axis also participates in the metastasis of pancreatic cancer cells by regulating lipid metabolism in tumor cells. Moreover, APOA1 overexpression can reduce tumor growth in vivo [23, 41]. Clinical studies have shown that abnormal ApoA1 levels in the serum or tissues of patients are associated with the prognosis of many types of tumors. In patients with breast cancer, the frequency of APOA1 copy number loss in tumor cells is negatively associated with serum ApoA1 levels. Lower ApoA1 levels were associated with a higher incidence of intraocular metastasis [20]. In patients with advanced non-small cell lung cancer, serum ApoA1 levels are associated with the frequency of EGFR T790M mutations and treatment response to EGFR tyrosine kinase inhibitors [21]. In patients with kidney clear cell carcinoma (KIRC), the expression levels of APOA1 mRNA in KIRC tissues were lower than those in para-cancerous and normal kidney tissues, and high expression of APOA1 mRNA in tissues was correlated with worse OS and disease-free survival. Moreover, the preoperative serum ApoA1 levels in patients with KIRC were significantly lower than those in healthy controls, and patients with KIRC with low serum ApoA1 levels had worse OS [24]. Serum ApoA1 level is an independent prognostic factor for OS in patients with CRC and advanced intrahepatic cholangiocarcinoma treated with PD-1 [22, 25]. Recently, Wang et al. found that low serum ApoA1 levels were associated with the PFS and OS of patients with DLBCL but were not an independent prognostic factor for survival in a cohort of 307 newly diagnosed patients with DLBCL [11]. While Yu et al. found that low serum ApoA1 levels were independent poor prognostic factor for OS and PFS in a cohort of 105 patients with DLBCL [12]. Since research on serum ApoA1 levels in newly diagnosed patients with DLBCL and its possible prognostic significance is scarce, we sought to investigate these matters in a larger DLBCL population.

In this study, we found that the serum ApoA1 levels were lower in patients with DLBCL than in healthy individuals. Similar result was also found in the study by Yu et al. [12]. Thereafter, we identified the optimal cutoff value for serum ApoA1 level, accordingly stratified patients into the low ApoA1 and high ApoA1 groups, and analyzed the relationships between serum ApoA1 levels and clinical and laboratory parameters in these two groups. We found that patients with DLBCL with low serum ApoA1 levels had a higher percentage of male, more B symptoms, higher levels of LDH, poorer performance status, higher percentage of advanced stage, more cases in which there was more than one extranodal site, higher IPI, and a higher percentage of non-GCB subtypes. Interestingly, we also found that the incidence of relapse or refractory disease in patients with DLBCL with low serum ApoA1 levels was significantly higher than that in patients with DLBCL with high serum ApoA1 levels. Moreover, Kaplan-Meier analysis demonstrated that low ApoA1 levels were associated with shorter PFS in patients with DLBCL, which was similar to the results of two previous study [11, 12]. Since the occurrence of relapse or refractory disease was associated with PFS, these results suggest that serum ApoA1 level may be a possible biomarker of treatment response and helpful for the choice of therapeutic regimens. Furthermore, multivariate analysis demonstrated that an ECOG score ≥ 2, LDH > ULN, non-GCB subtype, and absence of rituximab were independent poor prognostic factors for OS and PFS in patients with newly diagnosed DLBCL. Ann Arbor Stage III–IV and more than one extranodal site were independent poor prognostic factors for PFS, while low serum ApoA1 levels were independent poor prognostic factors for OS. This result is inconsistent with that found by Yu et al. [12]. Then we compared the clinical characteristics of patients with DLBCL between our cohort and Yu et al. cohort. We found that the sample size of Yu et al. cohort was so small. Furthermore, the percentage of patients with IPI > 2, or Ann Arbor Stage III–IV, or extranodal disease > 1, or ECOG ≥ 2, or LDH > ULN in their cohort was lower than us (26.7% vs. 40.0%, 58.1% vs. 66.0%, 14.3% vs. 34.0%, 6.7% vs. 21.1% and 40% vs. 49.7%), respectively. We think that the differences in clinical characteristics between these two cohort account for these inconsistent results, too.

Nevertheless, this study had some limitations. The selection bias from the retrospective analysis limited the efficacy of our results. Because the detection methods of ApoA1 in multiple centers were different, the optimal cut-off value of serum ApoA1 from our study may not be applicable for prognostic analysis in other centers [14, 20]. Since there were too many missing data on cytogenetic or molecular biological information in our cohort, we were unable to reveal the correlation between serum ApoA1 levels and the biological tumor characteristics of patients with DLBCL. Although serum ApoA1 levels are associated with treatment response in patients with DLBCL, the role of the APOA1 gene in the development and progression of DLBCL and its underlying mechanisms have not been confirmed. Previous studies demonstrated that ApoA1 has been shown to be involved in chemoresistance and metastasis in some types of solid tumors, and ApoA1 mimetic peptides had been confirmed to have anti-tumor effects [15]. It suggests that ApoA1 may be a potential target for DLBCL treatment. Therefore, prospective multicenter studies with comprehensive cytogenetic or molecular biological information on patients and related basic research are needed to elucidate the role of serum ApoA1 in the development and progression of DLBCL and its prognostic predictive efficacy.

## Conclusions

This study demonstrates the prognostic value of serum ApoA1 levels in patients newly diagnosed with DLBCL. Newly diagnosed patients with DLBCL with low serum ApoA1 levels showed more adverse clinical features than those with high serum ApoA1 levels. Low serum ApoA1 levels were associated with a higher incidence of treatment failure and inferior survival outcomes in patients with newly diagnosed DLBCL. These findings indicate that serum ApoA1 level may be a possible biomarker for predicting treatment response and survival outcomes.

## Data Availability

All data generated or analysed during this study are included in this published article.

## Abbreviations

DLBCL: Diffuse large B-cell lymphoma
NHL: non-Hodgkin’s lymphoma
IPI: International Prognostic Index
ECOG: Eastern Cooperative Oncology Group
LDH: Lactate dehydrogenase
ULN: Upper limit of normal
NCCN: National Comprehensive Cancer Network
ApoA1: Apolipoprotein A1
OS: Overall survival
PFS: Progression-free survival
ROC: Receiver operating characteristic
GCB: Germinal centre B cell
ABC: Activated B cell
